# Service evaluation of long acting buprenorphine subcutaneous injection (BUVIDAL) in the west Lothian community addictions service

**DOI:** 10.1192/bjo.2021.871

**Published:** 2021-06-18

**Authors:** Amy Martin

**Affiliations:** St. John's Hospital

## Abstract

**Aims:**

1. To establish if long acting buprenorphine subcutaneous injection retains patients in treatment.

2. To obtain the patient opinion of long acting buprenorphine subcutaneous injection and ascertain if it improved other aspects of their life for example relationships and employment.

**Method:**

Information was gathered from TRAK, the patient record recording system, and Illy, the prescribing system. This allowed data to be gathered on previous opiate substitute treatments and when the patient was commenced on the long acting buprenorphine injection. A patient questionnaire was used to obtain qualitative data on the patient's view of this treatment option.

**Result:**

West Lothian Community Addictions Service starting offering long acting buprenorphine injection as a treatment option in March 2020. Since then there has been a consistent demand from patients to be commenced on this treatment. On 31st January 2021 39/53 (73.6%) of patients who had been commenced on long acting buprenorphine for 6 months had been retained on this treatment. Moreover, 3 patients were lost to treatment due to transfer to Her Majesty's Prison. Patients who were commenced on this treatment option were both new to treatment and those who had previously been difficult to retain on methadone or sublingual buprenorphine. The questionnaire supported the antidotal feedback that patients found this treatment option to be hugely beneficial.

**Conclusion:**

Long acting buprenorphine injection has been well tolerated by patients and there has been a clear demand for this treatment option from patients accessing the service. It appears that the clarity of mind, that is associated with buprenorphine, has not been a barrier to retention in treatment. We have found the retention rate of the patients on this treatment option has been higher than the median 6 month retention for either methadone or buprenorphine, compared to a recent systematic review. In addition, it has helped patients consider employment, improve relationships and maintain a level of stability that they may not have previously achieved on either methadone or sublingual buprenorphine.

